# Differentiating the effect of social enterprise activities on health

**DOI:** 10.1016/j.socscimed.2018.01.042

**Published:** 2018-03

**Authors:** Bobby Macaulay, Micaela Mazzei, Michael J. Roy, Simon Teasdale, Cam Donaldson

**Affiliations:** a Yunus Centre for Social Business and Health, Glasgow Caledonian University, Glasgow, UK; b Glasgow School for Business and Society, Glasgow Caledonian University, Glasgow, UK

**Keywords:** Scotland, Social enterprise, Public health, Health intervention

## Abstract

An emerging stream of literature has focused on the ways in which social enterprises might act on the social determinants of health. However, this previous work has not taken a sufficiently broad account of the wide range of stakeholders involved in social enterprises and has also tended to reduce and simplify a complex and heterogeneous set of organisations to a relatively homogenous social enterprise concept. In an attempt to address these gaps, we conducted an empirical investigation between August 2014 and October 2015 consisting of qualitative case studies involving in-depth semi-structured interviews and a focus group with a wide variety of stakeholders from three social enterprises in different regions of Scotland. We found that different forms of social enterprise impact on different dimensions of health in different ways, including through: engendering a feeling of ownership and control; improving environmental conditions (both physical and social); and providing or facilitating meaningful employment. In conclusion, we highlight areas for future research.

## Highlights

• Enhanced understanding of the effects of social enterprise activities on health.
• Wider range of stakeholders consulted.
• Social enterprise understood as a complex and heterogeneous set of organisations.
• Unpacked how different types of social enterprise might vary in health impact.

## 1 Introduction

Understanding the role of organisations that work outside of formal health systems – within civil society, for example (Borzaga and Fazzi, 2014, Giarelli et al., 2014) – but which act to address factors in the social environment that favour or harm health, has been the subject of significant focus in recent times. Social enterprises – organisations that trade in the market in order to address one or more aspects of social vulnerability, particularly in local communities (Galera and Borzaga, 2009, Haugh, 2007) – have been the focus of particular attention, with a body of literature starting to emerge which considers the health and/or well-being impact of social enterprise-led activity. Rather than focusing attention on the role that social enterprises can play in providing health and social care products or services directly (Calò et al., 2017, Hall et al., 2015, Hall et al., 2012, Millar, 2012, Millar et al., 2016), this literature suggests that through addressing social vulnerabilities (Teasdale, 2010), social enterprises can improve individual and community health through acting on social determinants of health (Dahlgren and Whitehead, 1991, Solar and Irwin, 2010). This may involve providing or facilitating employment, developing social networks and connectedness; and/or improving the social and environmental conditions of the community. In essence, “creating spaces and conditions under which the empowerment of disadvantaged communities can become a reality” (Solar and Irwin, 2010, p. 22).

Our study builds upon this emergent stream of literature which focuses on the role of social enterprise in addressing the social determinants of health: the conditions in which people are born, grow, live, work and age, which are shaped by the distribution of money, power and resources at the global, national and local levels (Commission on Social Determinants of Health, 2008, Wilkinson and Marmot, 2003). Previous research ranges from grey literature written by practitioners (Boswell et al., 2009, McDermid et al., 2008, Westwater, 2009), to a growing body of (mainly conceptual) work by academics (Donaldson et al., 2011, Farmer et al., 2016, Macaulay et al., 2017, Roy et al., 2014, Roy and Hackett, 2017). Recently developed conceptual models (Macaulay et al., 2017, Roy et al., 2014, Roy et al., 2017a, Roy et al., 2017b) employ tools and methods common in the development and evaluation of complex public health interventions (viz. Craig et al., 2008) to identify processes and causal pathways between certain social enterprise-led activities and potential impacts on different dimensions of health and wellbeing. These range from aspects of physical and mental health, to social determinants such as income, education, housing quality, employment and working conditions, to associated aspects such as self-confidence, social connectedness and empowerment.

There remain, however, some limitations to this nascent field of study. Firstly, the majority of this literature has drawn primarily upon on the views of social enterprise leaders – the heads of organisations – and thus has not taken sufficient account of the views of beneficiaries/service users, staff and members of the broader community. Secondly, this literature has inadvertently reduced and simplified what is, in reality, a complex and heterogeneous set of organisations to what might appear as a relatively homogenous social enterprise concept. With these considerations in mind, we sought to understand: *whether, how and for whom different types of social enterprise-led activities affect health and wellbeing*. We utilised data gathered from in-depth qualitative case studies with three social enterprises in different regions of Scotland, and explored the health effects of social enterprise activities, the recipients of those effects, and the pathways involved in generating health impacts. While we consider that the findings of this study partially corroborate the potential of social enterprise as an alternative ‘upstream’ provider of community health (Farmer et al., 2012, Roy et al., 2013), the primary contribution of our study is to begin to unpack how different types of social enterprise might impact on (different dimensions of) health in different ways.

## 2 Methods

This qualitative study of three social enterprises was carried out through conducting in-depth semi-structured interviews with a broad range of stakeholders with knowledge of each organisation and the community in which they operate. Three well-established social enterprises were purposively sampled (Mason, 1996) to reflect a variety of social missions and governance structures. All of the case study organisations were based in one country – Scotland – which politicians claim is ‘the most supportive environment in the world for social enterprise’ (Roy et al., 2015) in cognisance of the difficulties involved in comparing social enterprises from different legal, political or socioeconomic backgrounds (Kerlin, 2010).

Five stakeholder groups were identified as being of interest to the research, due to their perspectives on the work and effects of the social enterprises:

1. Service users. The individuals whose benefit is the primary ‘social mission’ of the organisation and thus represent the intended focus for the social impact of the organisation.
2. Leaders of organisations. This group includes managers, directors and/or board members responsible for the overall governance and running of the organisation and who were able to provide an overview of the strategic aims and objectives of the organisation.
3. Staff members. This group includes those employed on various projects within the social enterprise and spent the most time with service users and the wider community.
4. Community Stakeholders. This group refers to those professionals who worked (and often lived) in the same geographical region, and were aware of the work of the sample organisations, and their impacts, in a professional capacity.
5. National Stakeholders. This includes those working in a professional capacity for organisations or bodies with a direct knowledge of the social enterprise and/or public health provision across Scotland (see Table 1).

A total of twenty-eight interviews were conducted, as well as one focus group of six participants (see Table 2 for full list). The interviews were conducted by the first author between August 2014 and October 2015. Ethical approval was granted by the University's Research Ethics Committee.

**Table 1:** Details of sample social enterprises.

	Type	Location	Social Purpose	Primary Activities
Case Study 1	Work-Integration Social Enterprise (WISE)	Small town in northern Scotland	To provide employment opportunities to those excluded from mainstream employment	Employs physically and mentally disabled adults in a variety of retail outlets and service roles.
Case Study 2	Community development-based	Rural community in the east of Scotland	To reverse economic and social decline in a fragile community, following the demise of the largest industry and employer.	Provides support and consultancy for small businesses; training and educational opportunities for people of various ages and abilities.
Case Study 3	Community-owned housing cooperative	Periphery of a large city in the West of Scotland	To improve the lives and conditions of the local residents of a relatively deprived area.	Provides affordable housing and other facilities including outdoor activities for children and a community hub.

**Table 2:** Sample social enterprises and number and type of respondents.

	Community Stakeholders	Social Enterprise Leaders	Social Enterprise Staff	Social Enterprise Service Users
Case Study 1	• Local NHS • Third Sector Interface • Economic Development	• General Manager	• Support worker • Assistant Manager	• Supported employees (×3)
Case Study 2	• Third Sector Interface • Local Council	• Managing Director	• Business support staff (×2) • Youth training support worker	• Small social enterprise leaders (x)2
Case Study 3	• Community Development Trust • Local Council • National Umbrella body	• Director	• Community Development Coordinator	• Management Committee (focus group)
National Stakeholders	• Public Health Researcher (×2) • Public Health Academic • Social Enterprise Umbrella Organisation (×2) • Social Enterprise Consultancy			

Health was only explicitly mentioned toward the end of each interview, where an open question was asked about the ways in which social enterprises could affect health, before probing to establish what aspect or facet of health participants were referring to. Such aspects included physical and mental health, as well as concepts such as wellbeing and emotional health. An ‘abductive’ approach was taken to the process of analysis and theory building (Eisenhardt, 1989, Thomas, 2010, Timmermans and Tavory, 2012), acknowledging participants' interpretations and building upon previous literature while allowing for new themes to emerge in an iterative manner. The data were initially coded by the first author using thematic coding (Saldaña, 2013), before being presented to co-authors for scrutiny and comment. Codes were then reconfigured in line with relevant theory and literature to explore the features that related to each case specifically. In order to facilitate this process, a series of matrices were populated and analysed through the Framework Analysis method (Ritchie et al., 2013) using the qualitative data analysis software QSR NVivo 10. Framework Analysis facilitates the consideration of large quantities of qualitative data belonging to numerous interrelated themes, allowing for the consideration of processes, outcomes and recipients to occur simultaneously. In accordance with the aims of the study, these were organised into several different categories: the activities and processes occurring within the social enterprises; the outcomes relating to health that were claimed to derive from those activities; and whose health was claimed to be affected. A number of social determinants of health were mentioned throughout the interviews, but were not considered relevant within this study unless explicitly linked to health outcomes by respondents. Through this analytical process, a number of key specific activities, processes and mechanisms utilised by the sample social enterprises were identified, namely: engendering feelings of ownership and control; improving environmental conditions (both physical and social); and providing or facilitating meaningful employment. The following section considers each of these themes in turn, also detailing the nature of the health effect, and the identity of the recipient of that effect.

## 3 Findings

### 3.1 Engendering feelings of ownership and control

A strong theme emerging from the data (but particularly explicit in relation to Case Study 3 – the housing co-operative) was that of supporting people to take control over a variety of forces that affect the lives of individuals and communities. These elements can be broadly considered to be consistent with the concept of ‘efficacy’, whether individual or collective, both of which have been strongly linked to health outcomes for individuals (O'Leary, 1985) and communities (for example, see Teig et al., 2009).

Regarding the effect of collective efficacy on health, several respondents intimated that placing responsibility in the hands of the community (through the transfer of responsibility from a public body to a community-controlled social enterprise) had both the instrumental effect of improving the local area, and building the wellbeing of residents through the knowledge that they, collectively, have the capability to do so:

*“You just have to stand back and take a think about how that affects somebody's life, whereby they have that kind of responsibility for themselves, for their own community and that, again, much overused word just now, that empowerment that's going to give … that ability to control your own life and, indeed, not have it run by people who are remote from you … I don't think that's difficult to imagine that that has a benefit to people's life chances and therefore, probably their health.”*

Case Study 3- Community Stakeholder 1

One element credited for facilitating and encouraging this form of collective efficacy, and the ensuing health benefits, was the ownership structure of the housing co-operative, specifically referencing the control and resources that the management committee has to enact change:

*“It is a social enterprise because … you have all run it, made it what it is, to get the money from the rents, it's all re-invested, you talk with the tenants, plan and I think just that, making it such a good place to live, I think I have got a home and so much else, sort of health wise and physical wellbeing, just, it's such a good base to have a happy home, happy neighbourhood and I think it's such a success here and it's a great comparison with the two things sitting side by side.”*

Case Study 3- Service Users (Focus Group)

The comparison referred to in the above excerpt relates to the conditions faced in the neighbouring council-owned estate, where the social and material fabric of the community was considered to be in a significantly worse state (Case Study 3- Service Users (focus group); Community Stakeholder 3). Respondents claimed that the ownership arrangement of their housing estate contributed positively to their health and wellbeing through having a stake in the organisation's governance, as the quote below indicates:

*“If you get directly involved in the actual running of it, I think what it can do for your confidence which then has a knock-on effect, I think, to your health and wellbeing and your kind of belief in yourself, that you can do pretty much anything.”*

Case Study 3- Staff 1

There was also a perceived effect upon members of the community not actively engaged in the organisation's management, but who were part of a ‘strong’ community that had the power and capacity to make decisions for itself:

*“There's a sort of social mental health that comes from a community strength, community mental strength and a sense that it’s [their responsibility] to do it … rather than always waiting for the council to do it and then complaining when it's not done fast enough or quick enough or bright enough.”*

Case Study 2- Community Stakeholder 2

The above quotations indicate that ‘strong’ communities benefit not only those who are directly responsible for exercising that power, but also others within the local area who felt empowered to control aspects of their own lives (Browning and Cagney, 2002).

At an individual level, increased self-efficacy was generally considered beneficial to good health:

*“There's a fairly universally recognised human trait that making decisions for yourself in most situations is an empowering thing, it actually means that you can live the kind of life you want to live and that that is generally good for your health, physical and mental.”*

Case Study 3- Community Stakeholder 1

However, when considering the specific ramifications of increased responsibility and control, self-efficacy was also perceived in some ways as a ‘double-edged sword’. On the one hand, the increased control over the circumstances which surround their lives, and especially the services they receive, is perceived to be positive for individuals. For example, some commented on the benefits that service co-design had in improving the service *tout court* and consequently on the individuals involved, both as beneficiaries of the service, and the inherent impact of them having a say and a direct influence on their everyday life conditions:

*“It's about mental health involvement, it's about bringing people with mental health problems in, and getting them involved in service design, commenting on strategies, designing services in the local authority to make them better. It's kind of designing the services for yourself and so on, which is great.”*

National Stakeholder 6

It was also suggested that increased personal responsibility may actually increase stress levels (see Schönfeld et al., 2017), and thus have a negative effect on the health of service users:

*“If you are in charge and control of aspects of your working life, what an impact that can have on your stress levels. And I'm guessing this should be positive but I can see how it could be a bit negative as well”*

Case Study 3- Community Stakeholder 1

From a practical point of view, the ability of individuals or communities to facilitate this increased power and control was seen to derive from the nature of the relationship between the community, the social enterprise and political actors such as the local council. Respondents commented upon the effect of being able to access services within the social enterprise and have a direct connection with an individual there, rather than having to deal with the ‘nameless, faceless bureaucracy’ (Case Study 3- Service Users (Focus Group) that can sometimes be symptomatic of the public sector (similar to that observed by Seanor and Meaton, 2008):

*“You have the working partnership with Citizens Advice [Bureau] and big credit unions, Money Matters and different other various, sort of, agencies or external agencies, that come in here to offer advice. So if you're looking at the health and wellbeing side, then there's a whole number of things that are centred in here that's been brought in here and that helps the whole community”*

Case Study 3- Service Users (Focus Group)

### 3.2 Improving the environment

Respondents spoke of the effect of the surrounding physical environment on health. This included the superficial appearance of the community, the internal improvement and structural integrity of buildings; the level of estate management; and the feeling of safety in one's own home. This theme was dominated by respondents from Case Study 3 due to its specific purpose of improving the internal and external condition of the housing stock which the co-operative owns and manages. There was broad agreement among all stakeholder groups of the effect of such improvements on the physical health of the community, and how that has a knock-on effect on their mental health and wellbeing (Case Study 3- Community Stakeholder 3). For example, the leader of Case Study 3 commented:

*“It's an important physical aspect of developing property housing which mitigates against health difficulties in relation to asthma and all those bronchial elements connected with damp housing, all that stuff is really important. We also take a view that the mental health aspect of stress and living in an environment which is difficult, dangerous, chaotic is a huge health problem.”*

Case Study 3- Leader 1

A number of respondents commented upon how physical health issues caused by living in cold, damp, draughty accommodation, especially regarding breathing and bronchial issues, could also be detrimental to mental health. One of the ways in which this occurred was the effect on the mental health of parents or carers through not having to worry about their children suffering from such issues. One of the staff members of Case Study 3 disclosed:

*“I think people's lives have been made easier, I think people's lives have been made better which I think then makes them healthier. You are not having to worry about your child sleeping in a damp room, you do not have a child with asthma, you now all these things have a direct impact on people's health and we now take that for granted.”*

Case Study 3- Staff 1

Related to this ‘relief from worry’, the effective management of housing and the surrounding environment was seen to have an additional effect on mental health through the reduction of residents' stress, as the quote below indicates:

*“We also take a view that the mental health aspect of stress and living in an environment which is difficult, dangerous, chaotic is a huge health problem. In getting that management right is not necessarily about putting new roofs on or putting better windows in or putting better heating systems in … So we've got an absolutely crucial role there to play in terms of the health sense, in order to ensure that people stress levels on a mentally health perspective dissipate”*

Case Study 3- Leader 1

This sentiment was echoed by both the staff and those living in the housing estate, with the recognition that having a positive and pleasant social environment can reduce stress and improve people's general health:

*“It just lifts people out of stress - that's a killer. Not many people are worrying about going out in a scheme and just shutting their door and worrying if they have to go outside and all that, people can relax in their area … I think that must have boosted the health up mega”*

Case Study 3- Service Users (Focus Group)

‘Stress’ is repeated numerous times throughout the different stakeholder groups. It was argued that a more holistic view of housing services, which values the psychological aspects of building management as much as the physical, structural aspects, could improve mental, as well as physical, health. In terms of the external appearance of the estate, there was recognition of the effect that can have on stress levels and general wellbeing. This in itself was interesting as it implies that it is not necessarily just the individual experience of living in a house or flat which was important, but the wider community experience of living in a safe and pleasant area also has a bearing. One of the employees of Case Study 3 indicated:

*“It is a very, very well maintained estate. You know, the grass is always cut, there is never any vandalism, there are no broken windows. All these things support a better community and I think, a happier community, and a healthier community.”*

Case Study 3- Staff 1

This sentiment was echoed by residents themselves who connected physical and psychological elements in acknowledging how a more pleasant appearance to the community can affect health:

*“The security and the looks, you know, it's a physical thing, it's a mental thing, I think, knowing you can just settle, less worry, do you know what I mean, it's an easier life”*

Case Study 3- Service Users (Focus Group)

Case Study 1 also aimed to improve the social environment in which people with disabilities live, both in terms of the area in which the organisation operates, and at an individual level, as the following quote indicates:

*“There would have been a large number of people sitting at home or putting stress on their carers at home. There would certainly be a lot of people in difficult situations, either on their own or with carers who were getting no support at all. So [the area] would be a much worse place for sure. And a lot more stress and people in a really bad situation.”*

Case Study 1- Community Stakeholder 1

Echoing this sentiment, the leader of Case Study 2 spoke of the mental health effects of social interaction within a workplace setting, whether provided or facilitated through the organisation by way of training or support for establishing a small business:

*“So a lot of mental health benefits to it; other than being sitting in a house on a play station 24/7 actually interacting with other people in the workplace, so a lot of mental health issue benefits.”*

Case Study 2- Leader 1

Taking a broader view of a community of place, one of the residents of the community-owned estate (Case Study 3) commented that having a ‘happy’ community was seen to protect the potentially vulnerable and have an effect of the wellbeing of the entire area:

*“I think a lot of the stuff that you can do, sort of mental wellbeing, just a good environment to bring [children] up, not only for the older adults to live in … I think just that, making it such a good place to live, I think I have got a home and so much else, sort of health wise and physical wellbeing, just, it's such a good base to have a happy home, happy neighbourhood”*

Case Study 3- Service Users (Focus Group)

On a much broader scale, national stakeholders repeated this sentiment, claiming that a positive social network around an individual can help them cope with health issues, and potentially ease the burden on stretched public services.

*“That they have social supports, they have relationships with each other, mutual support from others that help them to manage those difficulties in their life rather than just turning to their GP, or turning to social work.”*

National Stakeholder 3

### 3.3 Providing or facilitating meaningful employment

The provision or facilitation of employment whether paid or otherwise, has been widely recognised as having a positive impact on the health and wellbeing of people and communities, so long as this work is meaningful (Coats and Lekhi, 2008). Case Study 1 – the Work Integration Social Enterprise (or WISE) – provides employment to those excluded from mainstream employment, while Case Study 3 employs local elected volunteers in the management of the social enterprise. Case Study 2 provides a number of routes into employment, including: support and funding to set up and expand new businesses and social enterprises; targeted sector-specific and vocational training in collaboration with industries seeking qualified employees; and mainstream employment which was often taken up by local people working within the social enterprise.

In its broadest sense, employment was perceived to be key to individuals being able to understand and recognise their own abilities, and receive the wellbeing benefits which accompany that recognition, as expressed by a Community Stakeholder from a public health background:

*“The fact that what you need to be successfully in employment isn't just about technically being able to do a job, it also takes a very holistic view of people's health and capacity and the fact that being validated through work in its broadest sense is part of wellbeing.”*

Case Study 1- Community Stakeholder 2

More practically, various facets of working activities and the workplace environment were credited with providing a range of benefits. Social interaction (Case Study 1- Leader 1), the provision of healthy food options (Case Study 1- Service User 3) and involvement in physical activity (Case Study 2- Leader 1; Case Study 1- Leader 1) were seen to benefit both physical and mental health, as reflected in one interaction with a work-integration service user, interpreted from sign language by a support worker:

*Interviewer: Some people have told me that working at [Case Study 1] can be good for their health, why do you think they said that?*

*Respondent: Yes, it’s good for health.*

*I: Why do you think it’s good for health?*

*R: Because we have food. And it’s hard work.*

*I: And how does the food help your health?*

*R: It helps give me energy.*

Case Study 1- Service User 3

The leaders of Case Study 2, meanwhile, implemented programmes and initiatives to improve the physical and mental health of employees, with the recognition that such programmes often lead to improvements in the efficiency and quality of the work produced:

*“It improves our work because we had that opportunity to get some fresh air. Which not everyone got, and everyone felt more energised as well. And people were talking about how they felt that they were sleeping better, when they got up in the morning they weren't having to drag themselves out of bed, when they were going home they weren't sitting on the couch. So all that obviously has an impact on their work”*

Case Study 2- Staff 1

The remuneration gained from employment was recognised as being a factor connected to health:

*“If you're unemployed, it's a lot harder to stay healthy if you've got no money”*

Case Study 2- Service User 1

An improved financial situation was seen to lead to mental health improvements, both intrinsically (e.g. no longer having to cope with the stress of precarious income) and instrumentally (e.g. people being better able to cope with financial shocks and/or afford relatively basic amenities, which then have a knock-on effect on their mental health), as the following response explains:

*“If you've got a budget to spend then you can probably afford better food; you can probably afford to have a holiday which you might not be able to, and again there are mental health benefits to that”*

Case Study 2- Leader 1

However, with regard to the effect it has on supported employees, it was considered by the leader of Case Study 1 that the act of receiving money was more important than the quantity of money itself. In this sense it was the feeling of being (re-)integrated into the world of work which was the main reason for the health benefit, rather than the relief from poverty *per se*:

*“We provide everybody here with a reimbursement for their volunteer time, it's a minimal reimbursement but it helps pay their transport or buy their lunch or whatever. A lot of the people that get that see that as a payment, see that as “This is what I get for the work that I've done”. They absolutely love that, you know, it's a big part of them feeling that they're in the workforce. And I think that thing about value and wellbeing and that feeling of self-worth that that gives people, that would be the worst thing that would be lost I think.”*

Case Study 1- Leader 1

This response indicates that the symbolic act of earning money in return for labour, almost regardless of the amount of the money involved, boosts self-worth and wellbeing for the employees in the social enterprise through the recipient feeling that they are being rewarded for their contribution, regardless of the financial value placed upon that, an observation made recently in a social enterprise context by Chan et al. (2016). A caveat to this however may be that the individuals concerned often either live with their parents or within care environments, where they are often insulated from financial pressures, thus giving money more of a symbolic importance, rather than an instrumental one.

For other employees (as opposed to those involved through work-integration), there is an acknowledgement of the effect on their own wellbeing of working for what they perceive to be an ethically sound organisation (see Kamerāde and McKay, 2015):

*“Like even working for a business that you care about, it builds your confidence, it improves your wellbeing, that kind of thing. So, there are the very soft outcomes that go with it as well. I think it has a positive impact on wellbeing because of the ethos working.”*

Case Study 2- Service User 1

However, an employee commented on the potentially detrimental mental health effects of working in a social enterprise, predominantly relating to uncertainties in institutional funding and employment security:

*“Funding tends to run from year to year. So when people in here start to get to December, January time and they are waiting a decision on their funding as to whether they have got a job in April, the mood comes down a wee bit”*

Case Study 2- Staff 1

This could therefore imply that when things are going well it is the non-financial elements which take precedence and lead to beneficial health effects, whereas in times of uncertainty or when funding is cut, those elements can play a much larger role in defining health outcomes (Kim and von dem Knesebeck, 2015).

Finally, in Case Study 1, where physically or mentally disabled adults were integrated into employment, a positive health effect on the parents/carers of the beneficiary was suggested, through being provided with respite:

*“Probably the major health benefits with [Case Study 1] would be the carers, having relief from caring duties”*

Case Study 1- Community Stakeholder 1

This may indicate that there are likely to be indirect or subsidiary health benefits generated by the social enterprise and enjoyed not only by the service user, but by their families and their wider communities (Farmer et al., 2016).

## 4 Discussion

This study set out to understand: *whether, how and for whom different types of social enterprise-led activities affect health and wellbeing*. We found that different types of social enterprise impact on different dimensions of health in different ways, including through: engendering a feeling of ownership and control; improving the (social and physical) environment; and providing or facilitating meaningful employment. We discuss each in turn.

### 4.1 Engendering ownership and control

The social enterprise literature gives little attention to the health effects of ownership and control of community resources upon individuals and communities, despite previous emphasis on the importance of participatory governance to the work of social enterprises (Pestoff and Hulgård, 2016) and public health literature connecting elements of power and empowerment to improved health outcomes (Phelan et al., 2010, Solar and Irwin, 2010), including in relation to health inequalities (McCartney et al., 2013). In this study we found that two of the three organisations engendered feelings of ownership and control: Case Study 2 assumed responsibility for delivering formerly council-run services while Case Study 3 was a community-owned housing cooperative. We found that those respondents who considered ownership and control as important to people's health represented every stakeholder group except the leaders of social enterprises, perhaps implying a lack of recognition of this element on their part. It was further noted that there was the potential for increased independence to increase stress and thus have a detrimental effect upon health (see Schönfeld et al., 2017).

### 4.2 Improving the environment

The importance of the surrounding natural and built environment is a focus for many social enterprises (Muñoz et al., 2015), particularly in the context of recent emergent thinking about social enterprises as ‘spaces of wellbeing’ (Farmer et al., 2016, Fleuret and Atkinson, 2007). We add to this literature by stressing the important role that social enterprises can play in maintaining the social environment, as well as maintaining safe and pleasant surroundings, which we know can also lead to improvements in physical and mental health. Over and above the importance of the physical environment and, in particular, housing, to health and well-being (which is well documented: see, for example, Thomson et al. (2009)), our study lends weight to the notion that the ownership structure and economic democracy exercised through some social enterprise forms, can also have an impact on health through enhancing a sense of collective efficacy. This was most obviously demonstrated in Case Study 3 – the housing co-operative. While the links between employee ownership and health have been tentatively explored in the past (e.g. by Erdal, 2012), this is a subject that deserves far greater attention in the future.

### 4.3 Facilitating employment

The provision of employment is a focus for many social enterprises. The health effects of social enterprise as an alternative to mainstream employment, particularly for people with mental illnesses or intellectual or physical disabilities, and/or as a gateway to either mainstream or supported employment for other vulnerable people most at risk of poor health outcomes, are starting to be better understood (Ferguson, 2013, Lysaght and Krupa, 2011, Roy et al., 2017a, Roy et al., 2017b). In this study we found that the meaning of work assumes different connotations depending on the type of organisation. For Case Study 1, work validates individuals and improves wellbeing, while the social interaction, healthy food and physical activity for those involved improve physical and mental health. For Case Study 2, wellbeing was increased through working for an ethically sound organisation. There were also dissenting perspectives: the turbulence, precariousness and uncertainty caused by contract-dependency (and other forms of income uncertainty) was sometimes considered to have a potentially negative effect on the health of social enterprise staff, whose jobs often depended on the winning of grants or contracts. It is, of course, important to recognise these (and likely other) potentially harmful elements when considering the health effects of social enterprise-led activity, and to contribute to our understanding of what is an often-acknowledged, but rarely researched field of enquiry.

## 5 Summary and conclusion

This study sought to contribute to the body of literature on the potential health effects of social enterprise activities through gathering the views of previously unrepresented stakeholders, such as service users, while embracing the complex, varied and heterogeneous nature of the social enterprise sector. The key contribution of our study was to unpack the heterogeneity of the social enterprise form, enabling us to identify that different types of social enterprise impact on different dimensions of health in different ways, not all of which are intentional. This offers a more nuanced contribution than previous studies, which have often treated social enterprise as a homogenous form. Furthermore, none of the health impacts identified in our study was common to all three cases. This more differentiated understanding of the effects of various social enterprise activities upon the health and wellbeing of intended beneficiaries, and other stakeholders, is something that should be incorporated into future studies, and should help to improve extant conceptualisations, such as those set out by Macaulay et al. (2017) and Roy et al., 2014, Roy et al., 2017a.

Nonetheless, our study has some limitations. First, the small sample size (three cases) was sufficient to identify that different types of social enterprise impact on health in different ways. However larger sample sizes which account for a greater degree of heterogeneity within the social enterprise population are necessary to begin to understand whether and how particular types of health benefit are intrinsic to particular types of social enterprise. Equally important, comparative studies between (different types) of social enterprise and other organisational forms operating in similar areas and fields are necessary to understand whether there is any comparative advantage accruing to social enterprise because of any unique organisational characteristics. For example, do jobs created through work integration social enterprises have a greater impact on health to those created through publicly owned and/or private companies? Secondly, our study was undertaken in a single country widely recognised as providing a supportive environment for social enterprise. Future comparative studies could address the extent to which context influences the health outcomes created through social enterprise. Finally, our study is limited in that it did not attempt to ‘measure’ health, or even to measure impact on the social determinants of health. While our study is one of the first to ask beneficiaries about the impact on their health, the numbers of beneficiaries interviewed per case was small. However the conceptual work we have undertaken can pave the way for future quasi-experimental studies that can measure the direct and/or indirect health benefits regarding the health outcomes derived from (different types of) social enterprises, compared with other types of organisation, whether in the private, third or public sectors.

## Funding

This work was supported by the Medical Research Council and the Economic and Social Research Council [grant number MR/L003287/1].
